# Incidence and risk factors for high-level BK viruria: a single center study in China

**DOI:** 10.1186/s12985-020-01460-5

**Published:** 2020-11-26

**Authors:** Rui Xiong, Haimin Ye, Zhujing Liu, Xinchang Li

**Affiliations:** 1 Medical College of Nanchang University, Nanchang, Jiangxi China; 2 Department of Organ Transplant, Jiangxi Provincial People’s Hospital Affiliated to Nanchang University, No. 152 Aiguo Rd, Nanchang, 330006 China

**Keywords:** Kidney transplantation, BK viruria, Risk factors, Incidence

## Abstract

**Background:**

BK virus allograft nephropathy is a serious complication after kidney transplantation, and the effect of pre-emptive intervention for high-level BK viruria has been verified, but protocols after kidney transplantation for early identification of high-level viruria are lacking.

**Methods:**

This was a single-center study. The clinical data of the kidney transplant recipients and their donors in our center from January 1, 2015 to December 31, 2018, were collected. The patients were divided into the high-level BK viruria group (Group A) and a non-high-level BK viruria group (Group B) according to the qPCR results of BK virus DNA loads in urine samples. Significant variables were screened out by univariate analysis, and then the results were incorporated into a multivariate logistic regression model to analyze the independent risk factors for high-level BK viruria.

**Results:**

A total of 262 recipients were included in the study. The incidence of high-level BK viruria was 13.4% (n = 35), and the median time of detection was 181 (range 91–1119) days. Univariate analysis showed that donor type ($\chi^{2}$ = 21.770, *P* < 0.001), history of ATG/ATG-F application ($\chi^{2}$ = 4.543, *P* = 0.033), acute rejection (AR) ($\chi^{2}$ = 8.313, *P* = 0.004) and delayed graft function (DGF) ($\chi^{2}$ = 21.170, *P* < 0.001) were related to high-level BK viruria. After the inclusion of the multivariate logistic regression model, the results showed deceased brain and cardiac donors (*P* = 0.032, OR = 3.927, 95% CI 1.122–13.746), AR (*P* = 0.022, OR = 4.709, 95% CI 1.253–17.697) and DGF (*P* = 0.001, OR = 6.682, 95% CI 2.288–19.518).

**Conclusions:**

Donation by deceased brain and cardiac patients, history of AR and DGF were independent risk factors for high-level BK viruria after kidney transplantation.

## Introduction

BK virus allograft nephropathy (BKVAN) is a severe disease caused by BK virus (BKV) infection or reactivation, which often impairs kidney function irreversibly and is more common in kidney transplant recipients [1]. The usual progression of infection begins with BK viruria and progresses to BK viremia, eventually leading to BKVAN. The importance of prevention is underscored by the lack of a specific treatment regimen for BKVAN. In 2013, the American Society of Transplantation [1] recommended starting intervention at the high-level BK viremia stage, but in a few studies, preemptive treatment of patients with BK viruria seemed to be more advantageous [2–4]. Early identification of high-risk patients plays an important role in prevention. Currently, reported risk factors are usually reported by a single center and mainly focus on the analysis of BK viremia, with large differences in results, which is confusing.

This study summarized the incidence of high-level BK viruria from 2015 to 2018 and analyzed its risk factors to identify patients at an early stage and provide treatment to prevent the occurrence of BKVAN.

## Patients and methods

### Patient groups

In this study, we retrospectively collected the data of kidney transplant recipients from January 1, 2015, to December 31, 2018, at Jiangxi Provincial People’s Hospital Affiliated with Nanchang University. This study was approved by the Ethics Committee of Jiangxi Provincial People’s Hospital (Serial No. 2015094). The patients were divided into two groups according to the different monitoring results of BKV DNA loads in urine after transplantation: high-level BK viruria (Group A) (BKV DNA loads ≥ E + 07 copies/ml) and non-high-level BK viruria (Group B) (BKV DNA loads < E + 07 copies/ml or no BKV DNA in urine samples). BKV evaluation was performed on the day before transplantation to ensure that all patients were BKV negative before transplantation. After transplantation, BKV monitoring was carried out for all patients according to our monitoring protocol. We included patients in group A when the detected BKV DNA load in urine samples was ≥ E + 07 copies/mL and there was no BK viremia. The patients included in group B had a urine BKV DNA load < E + 07 copies/ml or lacked a BKV DNA load. Similarly, we needed to exclude all patients with BK viremia. The exclusion criteria were as follows: 1. the patient was diagnosed with BK viremia; 2. no regular follow-up data were kept after the operation; 3. the patient died or lost the transplanted kidney during the study period.

### Monitoring protocols

We followed the monitoring protocols recommended by the guidelines published by the American Society of Transplantation [5]. Regular urine BKV monitoring was carried out for kidney transplant recipients after transplant, and the monitoring frequency was monthly until month 9, then every 3 months until 2 years, and once a year until 5 years. The urine BKV DNA loads were detected by quantitative polymerase chain reaction (qPCR), and the plasma BKV DNA loads were detected when the urine BKV DNA loads ≥ E + 07 copies/ml. The qPCR detection instrument was the AGSAFD-9600 from the China Public Health (Shanghai) Biotechnology Co., Ltd., in Shanghai, China (detection threshold > 2000 copies/mL); the reagent kit was the BK Virus Nucleic Acid Detection Kit (PCR fluorescent probe method) from SINOMD Gene Detection Technology Co., Ltd., in Beijing, China.

### Data collection

The clinical data of all the recipients and their donors were collected, including donor factors (sex, age, BMI, renal type, serum creatinine before organ obtainment, left or right kidney), recipient factors (sex, age, BMI, preoperative dialysis method, ATG/ATG-F medical history, history of AR, DGF, infection 30 days after surgery, immunosuppression maintenance protocols, number of transplants, BK virus DNA loads within urine, time of high-level BK viruria), and immune factors (HLA mismatching points, cold ischemia time, warm ischemia time).

### Statistical analysis

The statistical analyses were performed with IBM SPSS software, version 25.0 (Armonk, NY, United States). The results of continuous variables were expressed as the mean ± standard deviation (SD) or median (interquartile range, IQR) and numerical (percentages) values were used for categorical variables. The chi-square test and the independent sample T-test were used for the single-factor comparison between the two groups. In the single factor analysis, the interaction between the factors was not excluded. To avoid missing meaningful variables, the results with a *P* < 0.1 were selected to enter the multivariate analysis. The results of univariate analysis (*P* < 0.1) were included in the multivariate logistic regression model analysis, and the forward LR method was used to screen the variables and eliminate the unintentional variables. *P* < 0.05 was considered to indicate statistical significance.

## Results

### Demographics and clinical characteristics of recipients and donors

**Fig. 1:**
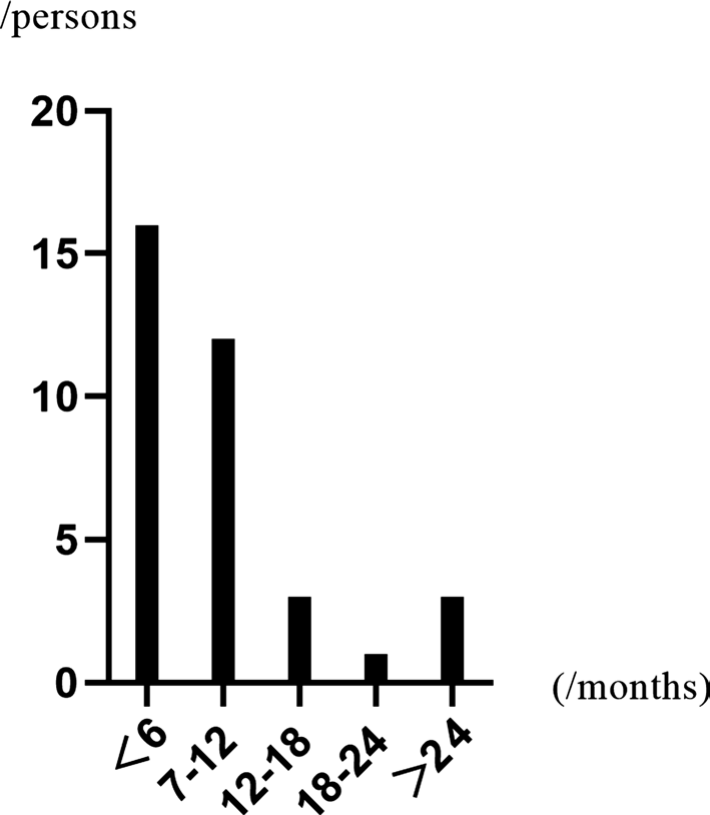
The time distribution of disease after kidney transplantation. The horizontal axis represents the time after transplantation, and the vertical axis represents the number of cases. This graph shows the highest incidence within 6 months of the transplant; it decreases over time and then increases again 2 years later

A total of 262 patients were included in this study, including 35 in group A and 227 in group B. All recipients were negative for panel reactive antibody and complement-dependent cytotoxicity before transplant. The demographic and clinical characteristics of all subjects are shown in Table 1. The prevalence of high-level BK viruria was 13.4% (n = 35), and the median time of detection was 181 (range 91–1119) days. Figure 1 shows the distribution of the onset time of 35 patients with high-level BK viruria. The median follow-up time for all recipients was 1004 (range 372–1954) days.

**Table 1:** Demographics and clinical characteristics of recipients and donors

Variates	All recipients n = 262	Group A n = 35	Group B n = 227	$\chi^{2} or t$ $value$	*P* value
Donors					
Sex (n, %)					
Male	197, 75.2%	28.80%	169, 74.4%	0.501	0.479
Female	65, 24.8%	7.20%	58, 25.6%		
Age (n, %)					
< 18	27, 10.3%	3, 8.6%	24, 10.6%	0.618	0.734
18–59	232, 88.5%	32, 91.4%	200, 88.1%		
≥ 60	3, 1.1%	–	3, 1.3%		
BMI (n, %)					
< 18 kg/m^2^	27, 10.3%	3, 8.6%	24, 10.6%	0.873	0.832
18–23.9 kg/m^2^	161, 61.5%	24, 68.6%	137, 60.4%		
24–27.9 kg/m^2^	64, 24.4%	7.20%	57, 25.1%		
≥ 28 kg/m^2^	10, 3.8%	1, 2.9%	9, 4.0%		
Donor type (n, %)					
Live	40, 15.3%	4, 11.4%	36, 15.9%	21.7	< 0.001
Brain death	12, 4.6%	1, 2.9%	11, 4.8%		
Cardiac death	138, 52.7%	9, 25.7%	129, 56.8%		
Brain-cardiac death	72, 27.5%	21, 60.0%	51, 22.5%		
Serum creatinine (mean ± SD)	93.2 ± 54.2 μmol/L	94.5 ± 65.2 μmol/L	93.0 ± 52.5 μmol/L	− 0.152	0.880
Left or right kidney					
Left	141, 53.8%	19, 54.3%	122, 53.7%	0.004	0.952
Right	121, 46.2%	16, 45.7%	105, 46.3%		
Recipients					
Sex (n, %)					
Male	189, 72.1%	22, 62.9%	167, 73.6%	1.731	0.188
Female	73, 27.9%	13, 37.1%	60, 26.4%		
Age (n, %)					
< 18	2, 0.8%	–	2, 0.9%	0.365	0.833
18–59	254, 96.9%	34, 97.1%	220, 96.9%		
≥ 60	6, 2.3%	1, 2.9%	5, 2.2%		
BMI (n, %)					
< 18 kg/m^2^	36, 13.7%	3, 8.6%	33, 14.5%	3.026	0.388
18–23.9 kg/m^2^	187, 7 1.4%	28.80%	159, 70.0%		
24–27.9 kg/m^2^	36, 13.7%	3, 8.6%	33, 14.5%		
≥ 28 kg/m^2^	3, 1.1%	1, 2.9%	2, 0.9%		
Dialysis (n, %)					
Hemodialysis	209, 79.8%	25, 71.4%	184, 81.0%	2.382	0.304
Peritoneal	51, 19.5%	10, 28.6%	41, 18.1%		
Others	2, 0.8%	–	2, 0.9%		
ATG/ATG-F (n, %)					
No	176, 67.2%	18, 51.4%	158, 69.6%	4.543	0.033
Yes	86, 32.8%	17, 48.6%	69, 30.4%		
AR (n, %)					
No	217, 82.8%	23, 65.7%	194, 85.5%	8.313	0.004
Yes	45, 17.2%	12, 34.3%	33, 14.5%		
DGF (n, %)					
No	215, 82.1%	19, 54.3%	196, 86.3%	21.170	< 0.001
Yes	47, 17.9%	16, 45.7%	31, 13.7%		
Infection within 30 days after surgery (n, %)					
Pulmonary infection	14, 5.3%	3, 8.6%	11, 4.9%	3.936	0.140
Urinary tract infection	14, 5.3%	4, 11.4%	10, 4.4%		
Others	233, 88.9%	28.80%	205, 90.7%		
Immune factors					
Immunosuppressive maintenance regimen (n, %)					
TAC + MMF + Pred^a^	259, 98.9%	34, 97.1%	225, 99.1%	1.046	0.306
Others	3, 1.1%	1, 2.9%	2, 0.9%		
Number of kidney transplants (n, %)					
First	258, 98.5%	35, 100%	222, 98.2%	0.626	0.731
Second	4, 1.5%	–	4, 1.8%		
HLA mismatching(mean ± SD)	2.0 ± 1.1	1.9 ± 1.2	2.0 ± 1.0	0.745	0.457
Cold ischemia time (mean ± SD)	10.2 ± 4.7 h	9.8 ± 4.2 h	10.3 ± 4.8 h	0.555	0.576
Warm ischemia time (mean ± SD)	4.5 ± 1.3 min	4.8 ± 1.2 min	4.5 ± 1.3 min	− 1.466	0.149

These all main data we collected were included into this table, and the corresponding chi-square/t values and P-values are contained in the last two columns of the table

^a^Tacrolimus + mycophenolate mofetil + prednisone (Tac + MMF + Pred)

(This table was relevant to Results.)

### Results of univariate and multivariate analyses

**Fig. 2:**
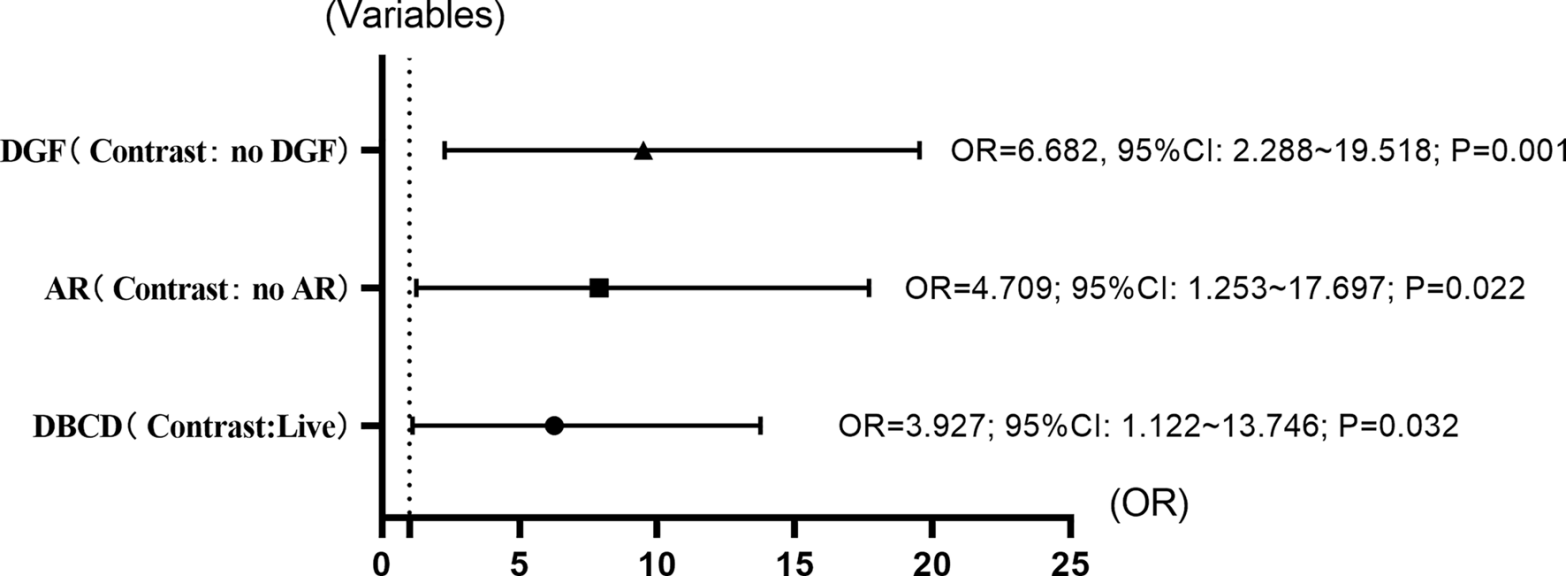
OR values for independent risk factors for high levels of BK viruria. The x-axis represents the value of OR, and the y-axis represents the type of variable. The figure shows the OR values, 95% confidence intervals and *P* values of the three independent risk factors. *DGF* delayed graft function, *AR* acute rejection, *DBCD* donation of brain and cardiac deceased

In Table 1, we present the univariate analysis results of different variables, and the variables with *P* value < 0.1 were input into the logistic regression model for multifactor analysis to identify the independent risk factors. The treatment history of ATG/ATG-F, AR, DGF and donor type were included in the regression model. The results showed the treatment history of ATG/ATG-F (OR: 0.339; 95% CI 0.084–1.370; *P* = 0.129), AR (OR: 4.709; 95% CI 1.253–17.697; *P* = 0.022), DGF (OR: 6.682; 95% CI 2.288–19.518; *P* = 0.001) and DBCD (OR: 3.927; 95% CI 1.122–13.746; *P* = 0.032) (Fig. 2).

## Discussion

First, we need to explain our selection of the BKV threshold. In 2004, a study showed that all active BKVAN showed BKV DNA load > E + 07 copies/ml of urine samples [6]. The guidelines published by the American Society of Transplantation in 2013 emphasize this result [1].

By monitoring the urine BKV DNA loads regularly after kidney transplant, we found that 13.4% of the patients (n = 35) were diagnosed with high-level BK viruria at the median time of 181 days in our center. In 2020, several scholars summarized the incidence of BK virus infection in Asia and found that the incidence of BK viruria after kidney transplantation was between 5.9 and 86.9% [7]. Although our data are also within this range, the wide range also affects the accuracy of these data. In fact, we must admit that the existing data were all reported by a single center, and there are too many uncontrollable factors leading to considerable differences in the results. Previously, some scholars also reported the prevalence of BKV infection among healthy people. Atonsson et al. [8] reported that the serum positive rate of BK virus in Australians was as high as 99% in people between 25 and 60 years old. Gossai et al. [9] investigated the prevalence of polyomavirus in the United States and found that the serum positive rate of BK virus was 87.6%. These reports reveal differences in the prevalence of BK virus infection in time and space. The highest incidence of BK viruria in renal transplant recipients was within 6 months of surgery, in accordance with other centers [10].

It is important to identify high-level BK viruria patients. Reischig et al. [11] have demonstrated graft damage from BK viremia in studies. Many centers, in fact, have attempted to treat high-level BK viruria. There is also a concern that there is no specific treatment for BKV infection in kidney transplant patients. The primary goal is usually to reduce the intensity of immunosuppression. We began to carry out preemptive intervention in 2015 to intervene in high-level BK viruria to prevent the occurrence of BKVAN, and the results were satisfactory. Among the 38 patients, BK viruria was effectively controlled in 32 patients (84.2%) within 1 year of treatment, and the remaining 6 patients (15.8%) also showed no infection progression, and no rejection reaction occurred in all patients after the immunosuppression intensity was reduced [The article is under submission]. Some researchers have also found that BK viruria also causes serum creatinine elevation by analyzing the survival of renal transplant recipients infected with BK virus [12]. Currently, the guidelines recommend high frequency BKV screening for all kidney transplant patients, which would undoubtedly result in significant medical costs. Early determination of a patient’s risk of infection can greatly save on medical costs and provide improved medical services to patients. Therefore, we conducted this study to explore the risk factors for high-level BK viruria.

As we know, no relevant studies have been reported internationally in this field, so we selected certain variables that may influence the occurrence of high-level BK viruria for analysis. The final results showed that DBCD, AR and DGF were independent risk factors for high-level BK viruria. AR and DGF were the expected results, and AR and DGF were also independent risk factors for BK viremia after renal transplantation [5]. However, DBCD surprised us. As is known, donor sources in China have undergone considerable changes in the twenty-first century. Influenced by traditional culture and religion, the development of DBD donors has been greatly hindered, which also severely limits the quantity and quality of our transplant work. Therefore, the criteria for donation in China were developed to solve the problem of the extreme shortage of donors in China. DBCD is the third type of donor in China (C-III), which is similar to category 4 in the Maastricht criteria [13]. Theoretically, we think that DCD might be one of the risk factors for the progression of BK virus infection. The incidence of DGF and primary nonfunction was significantly increased because DCD donors experienced hemodynamic disorders and the attack of underlying diseases. Generally, DBCD is similar to DBD, and the quality of the kidney is significantly higher than that of DCD. Therefore, DCD may be more closely associated with infection [5, 14]. These results are confusing. Whether the bias is caused by the small number of cases or the specificity of DBCD needs to be confirmed by more studies.

This study reports the risk factors for high-level BK viruria after renal transplantation, filling in the gaps in this field preliminarily, but there are also some limitations. In this single-center retrospective study, the insufficient sample size was still a limitation of its quality. Due to the limitation of objective factors, we could not compare all relevant factors. For example, there are limited types of immunosuppressive drugs that we use after transplant, induction therapy is not routine, donor-sourced BK virus surveillance has not been carried out, and so on.

## Conclusions

In the first 6 months after kidney transplantation, enhanced monitoring of the frequency of BKV infection is necessary, and DBCD kidneys and a history of AR/DGF are independent risk factors for high-level BK viruria. Therefore, we can use DBCD kidneys as a basis to identify patients early on and treat them. In future studies, prospective multicenter studies will always be an important direction in the field of BKV infection.

## Data Availability

The datasets used and analyzed during the current study are available from the corresponding author on reasonable request.

## Abbreviations

BKVAN: BK virus allograft nephropathy
BKV: BK virus
qPCR: Quantitative polymerase chain reaction
AR: Acute rejection
DGF: Delayed graft function
DBD: Donation of brain deceased
DCD: Donation of cardiac deceased
DBCD: Donation of brain and cardiac deceased
SD: Standard deviation
IQR: Interquartile range
Tac: Tacrolimus
MMF: Mycophenolate mofetil
Pred: Prednisone
OR: Odds ratio
CI: Confidence interval
